# Exploring Safety Outcomes Among Sanitary Workers in Punjab, Pakistan

**DOI:** 10.7759/cureus.81357

**Published:** 2025-03-28

**Authors:** Nadia Nisar, Nur Zakiah Mohd Saat, Dayana Hazwani Mohd Suadi Nata, Nurul Farahana Kamaluddin, Ismarulyusda Ishak, Ayesha Haque

**Affiliations:** 1 Community Medicine, National University of Sciences and Technology, School of Health Sciences, Islamabad, PAK; 2 Center for Community Health Studies (ReaCH), Faculty of Health Sciences, Universiti Kebangsaan Malaysia, Kuala Lumpur, MYS; 3 Environmental Health and Industrial Safety Program, Center for Toxicology and Health Risk Studies (CORE), Faculty of Health Sciences, Universiti Kebangsaan Malaysia, Kuala Lumpur, MYS; 4 Program of Biomedical Sciences, Center for Toxicology and Health Risk Studies (CORE), Faculty of Health Sciences, Universiti Kebangsaan Malaysia, Kuala Lumpur, MYS; 5 Anatomy, National University of Sciences and Technology, School of Health Sciences, Islamabad, PAK

**Keywords:** injuries, mental health, safety outcomes, sanitary workers, workload

## Abstract

Background: Sanitary work is the backbone of the municipal system. In Pakistan, it is performed manually, often under unhygienic conditions. As a result, workers are exposed to hazardous health and safety risks.

Objective: This study aimed to investigate the safety outcomes among sanitary workers in Punjab, Pakistan.

Method: A qualitative research approach was used, employing focus group discussions (FGDs) for data collection. Two FGDs were conducted, involving a total of 19 sanitary workers and two managers from the Cantonment Board Taxila and the Lahore Waste Management Company.

Results: Thematic analysis, conducted manually, revealed that while most sanitary workers reported minimal major injuries due to the consistent use of gloves and masks, they still experienced various minor health issues. These included skin allergies, burns, eye infections, respiratory infections, and minor cuts on the hands and other parts of the body. Additionally, participants highlighted significant psychological challenges, such as fear of chronic illness, stress, and depression, largely attributed to their demanding workloads.

Conclusion: The findings underscore the urgent need for improved occupational health policies in Pakistan to address the physical and mental health challenges faced by sanitary workers. The government should implement comprehensive health and safety regulations to enhance their well-being and mitigate the adverse health effects associated with their work.

## Introduction

Sanitation workers perform janitorial tasks such as cleaning, operating, maintaining, and managing solid and hospital waste [1,2]. This category also includes caretakers and toilet cleaners responsible for managing sewage and fecal waste treatment at disposal sites in domestic, institutional, and public settings [3]. Throughout their work, sanitation workers are exposed to hazardous gases, posing significant risks to their health. Studies indicate that inadequate waste management practices endanger both human health and the environment [4,5].

Data from public waste management organizations in Punjab, Pakistan, reveal that occupational safety and health (OSH) procedures are neither well-established nor implemented. These government companies were unable to provide documentation verifying their compliance with OSH standards. Research indicates that over 83% of sanitation workers employed by the Water and Sanitation Agency (WASA) rely on outdated equipment such as ropes, bamboo sticks, buckets, hoes, and picks to unclog sewage systems [6]. Consequently, workers at solid waste management facilities in several developing nations, including Pakistan, face severe health risks [6].

Sanitary workers form the backbone of municipal cleaning systems [1]. While sanitation processes in developed countries are largely automated, developing nations like India and Pakistan still rely heavily on manual labor, particularly in resource-limited metropolitan areas [7]. Due to the lack of modern sewage cleaning equipment, workers often enter underground sewerage systems through manholes to remove blockages manually. Many do so with minimal protective gear, using makeshift tools like spliced bamboo poles [8]. Each year, hundreds of workers in major cities across Pakistan and India lose their lives due to exposure to toxic gases and lack of oxygen while clearing sewage systems flushed with waste from millions of households, industries, and offices [1,9].

In Pakistan, particularly in Karachi, it is common for workers to enter main sewage pits without protective gear, such as gloves or face masks, to unclog blocked lines. These individuals, among the lowest-paid and most marginalized in society, endure living conditions that reflect their socio-economic struggles. Despite frequent exposure to occupational hazards and pathogenic microorganisms, most sanitary workers lack access to basic medical services [10].

Although previous studies have examined safety outcomes among sanitation workers, qualitative data on this critical issue remain scarce in the literature [6]. This study aimed to qualitatively explore the physical and psychological safety outcomes experienced by sanitary workers in Punjab, Pakistan, through focus group discussions (FGDs). The findings may provide valuable insights for policymakers in developing and implementing essential OSH policies, an area that has been largely overlooked in existing research [6].

## Materials and methods

This study employed a qualitative research methodology using FGDs for data collection. FGDs are particularly effective in exploring participants' experiences, attitudes, and complex behaviors [11]. The question guide was developed from the prior literature. The data collection process was conducted in multiple stages.

In the first stage, invitations were distributed to potential participants with the organization's approval. These invitations included informed consent forms and a brief overview of the research topic. Participants, including sanitary workers and managers, were reminded about the FGD sessions in advance. Workers with less than one year of experience were excluded, as their limited exposure to the field would not provide sufficient depth of understanding. Conversely, those with more than one year of experience were included, as their insights were considered more comprehensive regarding safety outcomes.

In the second stage, two focus groups were formed. Research suggests that 10-12 participants are ideal for an FGD session, as smaller groups risk being dominated by a single voice, whereas larger groups become difficult to manage [11]. Accordingly, this study organized one focus group with 10 participants from organization 1 and another with 11 participants from organization 2, as detailed in Table 1. Previous studies have also found nine to 12 participants to be suitable for FGDs [11]. The participants were selected using a convenient sampling technique. A few workers excused themselves to participate in FGDs due to their workload or other engagements. However, no incentive was offered to the participants.

**Table 1:** Participant details about the focus group discussions. FGD: focus group discussion.

Information	FGD 1	FGD 2
Organization	Organization 1	Organization 2
Number of participants	10	11
Number of organizers	One researcher, along with one helper for recording and one helper for other arrangements	One researcher, along with one helper for recording and one helper for other arrangements

In the third stage, the venue for the FGDs was determined. The venue should be neutral and conducive to open discussions, allowing participants to engage comfortably. Common FGD venues include school buildings, community centers, and health facilities, as they provide a neutral environment. For this study, the first FGD was conducted at the Cantonment Board Taxila, and the second at the Lahore Waste Management Company (LWMC). At both venues, seating was arranged in a U-shape, with participant name tags placed accordingly to allow the facilitator to address them by name, ensuring a structured and interactive discussion.

The data collection process consisted of the following steps. First, the researchers, accompanied by a research assistant and a note-taker, warmly welcomed the participants upon their arrival at the venue and guided them to their designated seats, arranged with name tags.

Second, the researchers provided a brief introduction to the FGD, explaining the purpose of the study and outlining the discussion process. They then initiated the conversation by asking the prepared questions in the local language (Urdu) to ensure clear understanding and effective communication.

Finally, after completing the FGDs, the researchers expressed their gratitude to the participants for their time and valuable contributions before concluding the session. It took 40 to 45 minutes to complete every FGD session.

After completing the FGDs, the audio-recordings were carefully reviewed, and the data were transcribed manually. The transcriptions were then translated from Urdu to English, ensuring that the original meanings and contextual nuances were preserved. However, the level of saturation was also ensured because the information started to repeat.

To verify accuracy, the translated English data were sent to an independent expert, who translated them back into Urdu. A cross-check comparative analysis was then conducted between the original Urdu transcriptions and the retranslated Urdu data. This triangulation process confirmed that the datasets were consistent in meaning, terminology, and intensity.

Following transcription and translation, themes and sub-themes were manually identified, and the analysis was performed accordingly.

Ethical considerations

The interview guide used for data collection was approved by the Universiti Kebangsaan Malaysia Research Ethics Committee, ensuring compliance with ethical research standards.

## Results

Table 2 presents the qualitative demographic data of the participants, including age, income, education level, and work experience.

**Table 2:** Demographic characteristics of the participants (sanitary workers).

Demographic profile		Frequency (F)	Percentage (%)
Age (years)			
	<20	3	14.28
	21-30	8	38.09
	31-40	6	28.57
	41-50	2	9.52
	51>	2	9.52
Income (Pakistani rupee)			
	<25000	4	19.04
	25001-35000	14	66.66
	35001>	3	14.28
Level of education			
	Uneducated	3	14.28
	Primary	9	42.85
	Matriculation	5	23.80
	Graduation	4	19.04
Experience (years)			
	1-5	7	33.33
	6-10	11	52.38
	>10	3	14.28

The findings reveal that the largest proportion of participants (38.09%) were aged between 21 and 30 years. A majority (66.66%) reported a monthly income ranging between 25,001 and 35,000 Pakistani rupees. In terms of education, 42.85% of participants had completed primary education. Additionally, the data show that 52.38% of participants had between six and 10 years of work experience, indicating a workforce with substantial industry exposure.

Safety outcomes among sanitary workers

The FGDs provided critical insights into the safety outcomes of sanitary work. This study identified three key sub-themes related to safety outcomes, including major safety hazards, skin allergies and diseases, and psychological issues, as presented in Table 3.

**Table 3:** Theme and sub-themes of safety outcomes.

Theme	Sub-themes	Results
Safety outcome among sanitary workers	Major safety hazards	No major injuries due to the use of safety gloves and masks
	Skin allergy and other infections	Faced skin allergy, skin burn, eye allergy, respiratory infections, and minor cuts
	Negative psychological outcomes	Stress, depression, social stigma, fear of chronic illness

Under the major safety hazards sub-theme, participants reported that the use of safety gloves and masks was effective in preventing major injuries. However, despite these protective measures, workers still experienced various health issues. Commonly reported conditions included skin allergies, such as rashes, burns, and eye irritations, as well as respiratory infections and minor cuts sustained during their work.

In the negative psychological outcomes sub-theme, participants highlighted significant mental health challenges associated with their profession. This included stress, depression, and the social stigma attached to their work. Additionally, many expressed a widespread fear of developing chronic illnesses due to prolonged exposure to hazardous working conditions.

Major safety hazards

Given that sanitary workers are frequently exposed to a wide range of hazards, ensuring effective safety measures is crucial for both their well-being and public health. Achieving positive safety outcomes is essential to prevent accidents, injuries, and the spread of diseases.

This study highlights that, despite the implementation of safety measures, including the provision of personal protective equipment (PPE), no severe injuries have been reported in recent years. One manager emphasized the effectiveness of these measures, stating:

"Usually, our workers never go through any major incidents because of sufficient personal protective equipment (PPE) and safety measures." (M1, FGD 1, 2)

However, risks remain despite the availability of PPE. For instance, one sanitary worker shared a close encounter with workplace hazards:

"Once, I found a snake at work but remained safe because I wore gloves and shoes." (W1, FGD 1)

Ensuring the consistent and proper use of PPE, such as goggles, gloves, and masks, is essential for protecting workers from potential dangers. Another worker shared an incident that underscored the importance of PPE:

"My gloves saved me from electric shock while digging out a hole." (W1, FGD 2)

These first-hand accounts highlight the critical role of PPE in preventing injuries and accidents among sanitary workers. Proper enforcement and adherence to safety protocols remain fundamental in safeguarding workers from workplace hazards.

Skin allergies and other diseases

Sanitary workers frequently suffer from skin allergies and respiratory illnesses due to constant exposure to hazardous gases, chemicals, and dust. Participants in this study reported experiencing skin rashes on their hands, body, and around the eyes. Many workers described these health issues as routine, with one stating:

"Skin rashes are widespread during work, but we also face significant issues such as skin burns, severe rashes, minor eye infections, and cuts on our hands." (W1, FGD 1, 2)

Exposure to harmful gases presents another serious risk. One worker shared a distressing experience:

"While working in a drain, I always fear exposure to harmful gases. On one occasion, I lost consciousness due to the black water and the detrimental effects of the gases." (W1, FGD 1, 2)

Another worker further emphasized the health risks associated with waste management:

"Managing waste leads to frequent respiratory infections and skin allergies." (W1, FGD 1, 2)

These experiences highlight the urgent need for enhanced protective measures, including proper ventilation, improved PPE, and regular health check-ups, to safeguard workers from occupational health hazards.

Psychological issues

The demanding nature of sanitary work contributes to significant psychological stress, burnout, and other psychosocial challenges. Sanitary workers in Pakistan often face societal stigma, which fosters feelings of humiliation and social exclusion. All participants in this study expressed experiencing these negative emotions, stating:

"We have felt humiliation and embarrassment due to societal stigmatization. This negative perception leads to social exclusion and a constant feeling of being underappreciated and ignored." (W1, FGD 1, 2)

In addition to social stigma, the intense physical demands and heavy workload contribute to stress and depression. Both workers and managers strongly agreed with the following statement:

"The physical demands of sanitary work, combined with hectic schedules and a heavy workload, create severe stress and depression. We constantly feel overwhelmed by the pressure to meet productivity goals and deadlines. Sometimes, we also worry about the risk of developing chronic illnesses." (M1, W1, FGD 1, 2)

These accounts underscore the urgent need for mental health support, including workplace counseling, stress management programs, and social awareness initiatives to combat stigma and improve the psychological well-being of sanitary workers.

## Discussion

The findings of this study underscore the complex nature of safety outcomes in sanitary work. Safety outcomes encompass various risks associated with the profession, which this research categorizes into three subthemes: safety hazards, skin allergies and diseases, and psychological issues.

During FGDs, all participants reported that they had never experienced major injuries, attributing this to the availability of PPE. However, despite the implementation of protective gear such as gloves and masks, sanitary workers remain exposed to health risks. Many participants reported skin burns, rashes, eye infections, and minor cuts, aligning with previous research documenting similar challenges. Studies indicate that sanitation workers frequently encounter work-related risks such as sun exposure, cold air, sewage, infectious organisms, chemicals, animal excreta, and sharp objects, all of which contribute to dermatological disorders affecting the face, hands, feet, and other exposed skin areas [12,13]. Other research highlights that contact with chemicals and medical waste can result in skin disorders and additional health complications [14,15]. Additionally, wounds sustained on the job pose a serious risk of infections, including hepatitis, reinforcing the persistent threat of chronic illness among sanitation workers [2,16]. The manual nature of sanitation work in developing countries like Pakistan and India, as opposed to the mechanized systems in developed nations, exacerbates these health risks.

FGD data also revealed significant respiratory health issues among workers. Participants frequently reported respiratory infections and acute distress due to exposure to harmful gases and dust. These findings align with existing literature, which associates sanitation work with pulmonary tuberculosis, chronic obstructive pulmonary disease (COPD), bronchial asthma, reduced lung function, inflammatory mediator imbalances, and oxidative stress [17-19]. Studies further indicate that exposure to dust and toxic gases often leads to symptoms such as wheezing, coughing, and rhinitis [20,21]. These findings highlight the critical need for improved protective measures to mitigate respiratory health risks.

The psychological toll of sanitation work is also profound. Participants described experiencing stress, depression, and social stigma due to their profession. This aligns with broader research showing that societal stigma, particularly in Pakistan, where a large portion of sanitation workers belong to marginalized communities, leads to social exclusion and psychological distress [22,23]. Despite their crucial role in maintaining public health, sanitation workers often face neglect, discrimination, and even demonization, making them highly vulnerable to physical, psychological, emotional, and social harm [24].

The FGD findings emphasize the significant psychological burden sanitary workers endure, directly linked to the demanding nature of their job. Participants expressed stress and despair stemming from physically exhausting tasks, strenuous schedules, and overwhelming workloads. The pressure to meet productivity targets and deadlines further intensifies their anxiety and fear of chronic illness. Research suggests that sanitary workers frequently experience high levels of fear due to prolonged postures, heavy labor, confined spaces, and excessive workload, all of which heighten their mental stress [25]. Similar studies have indicated that strict deadlines and time constraints contribute to mental exhaustion among workers [26]. Supporting these findings, research demonstrates that excessive workloads can impair job performance and increase cognitive failure, which, in turn, raises the risk of workplace injuries [2]. Additionally, workplace stressors, including heavy workloads, time pressure, and conflicting job responsibilities, have been directly linked to cognitive failure, further exacerbating the risk of injuries among sanitary workers [27].

Implications

This research has several important implications. Firstly, the findings provide valuable insights for policymakers, supporting the development and implementation of occupational health policies aimed at reducing the adverse health effects experienced by sanitary workers. Effective policy interventions based on this research could significantly improve workplace safety and health standards in this critical sector.

Secondly, the study contributes to academic and research discourse by enhancing the understanding of the working conditions and challenges faced by sanitary workers in Pakistan. These insights can inform future studies and scholarly discussions on occupational health, particularly in developing countries with similar labor conditions.

Finally, to further validate and expand upon these findings, future research should adopt longitudinal and mixed-method approaches. Such studies would provide a more comprehensive understanding of the long-term health effects and assess the effectiveness of safety interventions over time.

Limitations

This study has several limitations that should be considered. Firstly, it employed a qualitative methodology with a relatively small sample size of 21 participants. While this approach provided in-depth insights, future studies could benefit from a mixed-methods approach, incorporating quantitative data to enhance the generalizability and robustness of the findings.

Secondly, the research was geographically limited to Punjab, Pakistan. To ensure a broader and more representative perspective, future research should include participants from other provinces, capturing regional variations in working conditions, safety measures, and occupational health risks across the country.

Finally, this study focused exclusively on sanitary workers and supervisors, overlooking perspectives from policymakers and key stakeholders. Future research should incorporate interviews with policymakers, industry leaders, and regulatory bodies to better understand policy implementation challenges and explore systemic solutions for improving occupational health and safety in the sanitation sector.

## Conclusions

This study concludes that the use of gloves and masks has been effective in reducing major injuries among sanitary workers. However, they continue to face various health challenges. Participants reported experiencing mild skin allergies, burns, eye infections, respiratory illnesses, and minor cuts on their hands and other exposed body parts.

Beyond physical health risks, the study also highlights significant psychological distress among workers, including stress, anxiety, and a persistent fear of developing chronic illnesses, largely due to the physically demanding nature of their job. These findings emphasize the urgent need for strengthened safety measures, improved occupational health policies, and psychological support programs to enhance the overall well-being of sanitary workers.
